# Book Reviews

**Published:** 1873-05-01

**Authors:** 


### Book Reviews.

The Sanitarian. This is a monthly journal of 48 pages, published in excellent style, by A. S. Barnes & Co., New York and Chicago. It is edited by A. N. Bell, M.D., of Brooklyn, N. Y.

The first number was issued in April, and the second has just come to hand. As its name imports, it is devoted entirely to Sanitary science in its liberal and proper sense. The field it occupies is a most important and useful one, and the editor admirably qualified for the work he has undertaken. The two numbers before us contain articles of more value to every intelligent reader than the subscription price for the year, which is $3.00. We hope the enterprise will be abundantly sustained.

Family Thermometry; A Manual of Thermometry, for Mothers, Nurses, Hospitals, etc., and all who have charge of the Sick and of the Young. By Edwin Seguin, M.D. New York: G. P. Putnam & Sons, Fourth avenue and Twenty-third street—1873. Price, 75 cents.

This is a very neatly published little 12mo. of 72 pages, received through the bookstore of Jansen, McClurg & Co., of this city. It is written in a familiar, yet interesting style, and is designed to afford that elementary instruction in regard to noting the temperature of the human system in health and disease, which is needed by all who have charge of the sick. It will be found a useful aid to the busy practitioner, as well as to the nurse.

Fourth Annual Report of the State Board of Health of Massachusetts. January, 1873. Boston: Wright & Porter, State Printers.

This is an unbound octavo volume, of 473 pages, containing the annual report of the State Board of Health, with accompanying documents, for 1872. The whole volume is filled with matter of great value. But the articles on Sewerage and Drainage, on the Use of Alcoholic Drinks, on Infant Mortality, on The Causes of Consumption, and on House Accommodation for the Poor in our most populous cities, are worthy of special attention. They show very clearly that great progress is being made in improving the sanitary condition, and consequently the happiness, of the more enlightened communities throughout the world. In doing this they reveal still more clearly the fact that other and far greater improvements are both attainable and necessary.

